# Patient-Facing Clinical Decision Support for High Blood Pressure Control: Patient Survey

**DOI:** 10.2196/39490

**Published:** 2023-01-23

**Authors:** David Dorr, Chris D'Autremont, Joshua E Richardson, Michelle Bobo, Christopher Terndrup, M J Dunne, Anthony Cheng, Robert Rope

**Affiliations:** 1 Oregon Health & Science University Portland, OR United States; 2 RTI International Research Triangle Park, NC United States; 3 Vanderbilt University Medical Center Nashville, TN United States

**Keywords:** high blood pressure, hypertension, clinical decision support, shared decision-making, blood pressure control, decision-making support, patient engagement, patient support tool

## Abstract

**Background:**

High blood pressure (HBP) affects nearly half of adults in the United States and is a major factor in heart attacks, strokes, kidney disease, and other morbidities. To reduce risk, guidelines for HBP contain more than 70 recommendations, including many related to patient behaviors, such as home monitoring and lifestyle changes. Thus, the patient’s role in controlling HBP is crucial. Patient-facing clinical decision support (CDS) tools may help patients adhere to evidence-based care, but customization is required.

**Objective:**

Our objective was to understand how to adapt CDS to best engage patients in controlling HBP.

**Methods:**

We conducted a mixed methods study with two phases: (1) survey-guided interviews with a limited cohort and (2) a nationwide web-based survey. Participation in each phase was limited to adults aged between 18 and 85 years who had been diagnosed with hypertension. The survey included general questions that assessed goal setting, treatment priorities, medication load, comorbid conditions, satisfaction with blood pressure (BP) management, and attitudes toward CDS, and also a series of questions regarding A/B preferences using paired information displays to assess perceived trustworthiness of potential CDS user interface options.

**Results:**

We conducted 17 survey-guided interviews to gather patient needs from CDS, then analyzed results and created a second survey of 519 adults with clinically diagnosed HBP. A large majority of participants reported that BP control was a high priority (83%), had monitored BP at home (82%), and felt comfortable using technology (88%). Survey respondents found displays with more detailed recommendations more trustworthy (56%-77% of them preferred simpler displays), especially when incorporating social trust and priorities from providers and patients like them, but had no differences in action taken.

**Conclusions:**

Respondents to the survey felt that CDS capabilities could help them with HBP control. The more detailed design options for BP display and recommendations messaging were considered the most trustworthy yet did not differentiate perceived actions.

## Introduction

### Overview

High blood pressure (HBP) is a common condition in the United States, affecting roughly 47% of adults [1]. Persistently elevated blood pressure (BP)—hypertension—is a primary predictive factor for heart disease and stroke, which are among the most common causes of death in the United States [2]. Despite its prevalence, hypertension often goes underdiagnosed and undertreated [3]. The evidence base for the benefits of identifying HBP and reducing it through behavioral and lifestyle changes, medications, and careful monitoring is strong [4]; adherence to recommendations remains less than 50% for the population overall, and BP control has worsened through the COVID-19 pandemic, especially for vulnerable populations [5,6].

### Significance

As part of a project to develop an effective patient-facing CDS tool for hypertension management, we needed to understand how best to engage and motivate patients to use this tool through behavior science by understanding the knowledge, attitudes, and anticipated responses of potential patients to CDS systems.

### Background

There are many challenges to controlling HBP, especially when it is diagnosed as hypertension. First, hypertension is known as the “silent killer” [7] as elevated BPs are asymptomatic, leading to a lack of engagement from patients. Second, measuring BP requires frequent measurements and attention to protocol to assess control; home BP monitoring is frequently recommended yet rarely followed, leading to uncertainty about control and increased risk of adverse events from overtreatment [8]. Third, the therapeutic index in controlling BP can be narrow; a large BP trial, the Systolic Blood Pressure Intervention Trial [9], showed a 25% relative reduction in cardiovascular events in the tightly controlled BP group compared to that in the less intensive group (<120/80 vs 140/90 mm Hg, respectively) but a substantial increase in adverse events such as dizziness, falls, electrolyte disturbances, and acute kidney injury. Lastly, and perhaps most pressing, the role of the patient is crucial in BP control: behavioral and lifestyle changes can reduce BP by more than 15 mm Hg in most patients [10]. Given that most people lack symptoms for HBP, patient engagement and motivation remain a substantial issue.

An understudied area is taking recommendations from CDS and making them patient-centered. Work in patient-centered CDS explores the best way to engage patients beyond self-management support, sharing and translating recommendations and providing them directly to patients [11]. A patient-facing tool with robust CDS—providing the right information at the right time in the right format through the right channel [12]—may afford a way to better help patients both self- and comanage their BP and related conditions [13]. Encouraging patients to set goals (eg, smoking cessation, physical activity, diet and salt or sodium intake, weight, and alcohol intake), recognize when medications may be of help, and recognize adverse events can promote patient agency and engagement in BP management while also helping their care teams to obtain a more complete understanding of the patient’s cardiovascular health [14,15].

Goals, treatment preferences, and personal priorities may vary considerably, making recommendations difficult to implement. Assessing patient perceptions of priorities for goal setting is critical for designing CDS tools for engaging patients in treating HBP. Moreover, engaging people to set and follow goals requires behavioral change: behavior science has both cognitive precepts such as self-efficacy and behavioral economics concepts such as choice architecture, structured incentives, prosocial messaging, and social trust that may improve motivation and engagement [16,17]. Choice architecture is the ordering of options or defaults to help people make decisions more easily [18] and structured incentives—such as loss avoidance—help maintain motivation [19]. Social trust may be enhanced through well-sourced information and clinician recommendations [20]. Prosocial messaging encourages people to consider the beneficiaries of their behaviors when changing behaviors [21]. Focusing on others can be strong motivation: a review of older adults and people making changes after heart attack or stroke showed that team-based engagement with challenges and achievements were more effective in encouraging healthy behaviors [22-25]. However, studies of behavior science to guide CDS, especially with patients, are limited and results are mixed despite their promise [26].

The purpose of this study was to examine perspectives and experiences of people diagnosed with hypertension, particularly around health literacy, self-management strategies and other treatments, and general attitudes toward shared decision-making and CDS tools.

## Methods

### Overview

This work had two phases: (1) survey-guided interviews with a limited cohort and (2) a nationwide web-based survey. Participation in each phase was limited to adults aged between 18 and 85 years who had been diagnosed with hypertension.

### Ethics Approval

The Oregon Health & Science University’s institutional review board (IRB) approved this study (STUDY00020522).

### Phase 1: Guided Synchronous Interviews—Development and Recruitment

We recruited English-speaking participants from internal medicine patients at a primary care clinic. Initial contact was made via email, in which patients were told that the focus of this study was to assess attitudes and preferences for hypertension treatment. Participants were consented to record the interview and to a review of their medical record to identify medications and BP measurements. Interviews were conducted over 30-60 minutes via videoconference with screen sharing. Participants were given the opportunity to ask questions and make additional comments throughout the interview.

The phase 1 interview questions were derived from the 5 rights of CDS, attempting to help identify the right information, person, format, channel, and time, essentially attempting to understand patient perception of their role in the process [27]. In addition, we used cognitive and behavioral economics theories to drive questions, focusing on where choice architecture and social trust may be helpful in building the tool [22-25]. Topics in the phase 1 interviews included demographic questions and current HBP knowledge. We also sought to understand whether defaults could be set through questions about home monitoring and self-management strategies such as lifestyle changes, drawn directly from the guidelines, and general attitudes toward shared decision-making and CDS tools. Finally, patients were presented with recommendation-based case studies and asked what each patient should do: these were matched with questions asked of providers to understand alignment. The interview also used a modified version of the High Blood Pressure-Health Literacy Scale instrument to assess patient health literacy: this survey has a set of scenarios with structured and open-ended answers [28]. High literacy was defined as >80% correct, the top quartile from validation sample in the scale’s development. The interview questions were implemented as a Qualtrics survey and the interviewer filled out the survey during each session (Multimedia Appendix 1).

### Phase 2: Web-Based Patient Survey

A 10-minute web-based English language survey was deemed necessary to better identify generalizable trends. We developed the survey based closely on the interview guide, retaining many of the questions, albeit revising for clarity in the absence of an interviewer. The survey included questions that assessed goal setting, treatment priorities, medication load, comorbid conditions, satisfaction with BP management, and attitudes toward CDS (Multimedia Appendix 2).

We also included 3 sets of paired information displays, based on work by Shaffer et al [29] and the results of phase 1. The main goal was to assess whether questions with more information that enhanced social trust (with authoritative references) or provided clearer defaults were more likely to enhance trust and guide actions than those with less information. The displays were shown in randomized order and consisted of low (A) and high (B, ie, A/B testing) information tailoring, where low tailoring provided the recommendations within minimal additional information and high tailoring increased the amount of information overall [30].

**Figure 1:**
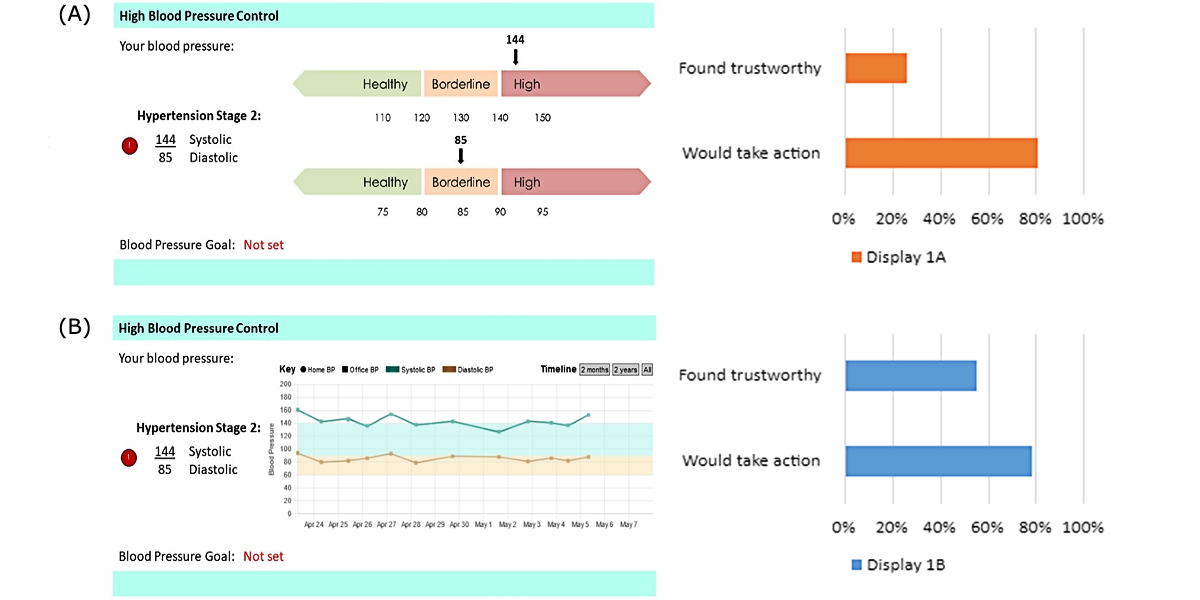
Visualizing the comparison of blood pressure history.

The first set of displays compared 2 options for representing the patient’s recent BP history. The first option (shown in the results shown in Figure 1) overlayed a current average BP (systolic and diastolic) onto colored bars representing ranges for healthy, borderline, and high; these were intended to simplify the choice to take action through choice architecture. The second option provided a line graph of the recent BP history with colored bands representing the healthy and borderline ranges; the additional data were intended to enhance trust by providing more data.

**Figure 2:**
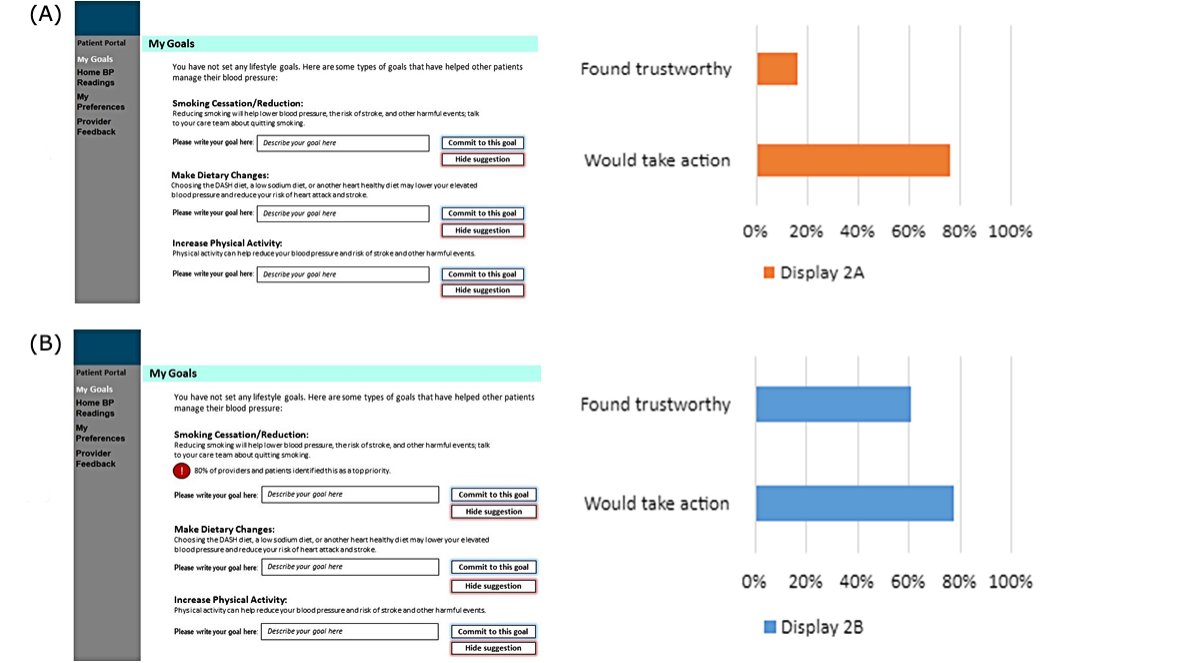
Lifestyle change goals with or without advice from clinicians regarding priorities.

The second set of displays (see results shown in Figure 2) compared suggested behavioral change goals with and those without added messaging around social norms. For example, a suggested goal of reducing smoking would have a higher amount of social trust enhancement with a message such as “80% of providers and patients identified this as a top priority.”

**Figure 3:**
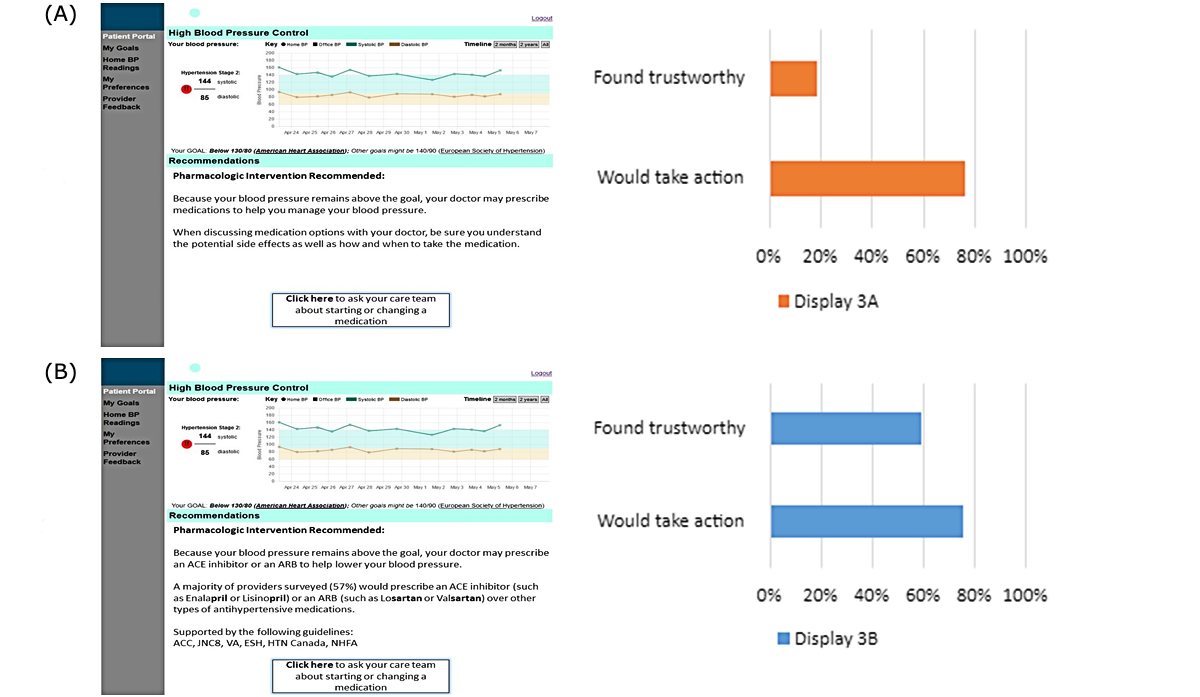
Potential hypertension medications with and without specific medications and supporting guidelines.

The third set of displays (see results shown in Figure 3) compared messaging around suggesting pharmacologic interventions. The low information tailoring option provided a simple message that one’s doctor may prescribe medications to help manage BP. The higher information option cited a survey of general provider preferences for particular drug classes and drug names and also referenced care guidelines supporting the pharmacologic option.

### Phase 2: Recruitment

With IRB approval, we contracted Qualtrics to gather 500 survey responses from patients aged between 18 and 85 years with a diagnosis of hypertension. IRB-approved recruitment language was provided to Qualtrics, and we incorporated their survey design expertise while finalizing our survey. Participants received credit toward a reward, administered by Qualtrics in accordance with their agreement with survey participants. Recruitment was stratified to include roughly equal proportions of male and female participants, as well as increased distributions from racial and ethnic minorities to collect a more generalizable sample set.

### Analysis

From the phase 1 survey–guided interviews, we summarized results using descriptive statistics, focusing on the most frequent and highest-priority responses. We also performed content analysis on the open-ended questions to understand the most common responses; these questions were largely focused on suggestions for display improvement or feedback about the HBP tool. Analysis was solely focused on extracting the ideas from each comment and summarizing them. For the phase 2 survey, we summarized results by frequency of answer selection. Likert scales were used for the paired visualizations requiring respondents to select one display as more trustworthy than the other, as defined by their own sense of trust, and the likelihood of taking action based on each display (actionability). Each used a 1-7–point range and the responses were summarized by the percentage of responses in the top 3 categories. Since trustworthiness was a dichotomous choice between 2 displays, the percentage selected was compared with a sign test. Categorical responses were compared with a chi-squared test for significant variations in response at the *P*=.05 level.

## Results

### Survey-Guided Patient Interviews

In total, 18 patients with hypertension were interviewed, out of 38 patients contacted, with 1 patient excluded from the analysis due to technical challenges preventing the interviewee from viewing the shared screen. Of the 17 remaining participants, summarized in Table 1, all identified as White, with a mean age of 69.2 years. This was a health literate group, with a mean modified HTN-HLS score of 84.9%, and 15 of 17 having scores of >80% (high literacy). All 17 participants reported having measured BP at home either currently or in the past. None were current tobacco smokers, though 7 (41.2%) reported having previously smoked. Only one patient (5.9%) was a heavy drinker. Overall, 35% of patients reported having atherosclerotic cardiovascular disease (ASCVD), although they did not remember having a heart attack or stroke. One (5.9%) participant reported a diagnosis of heart failure, 3 (17.7%) reported diagnoses of diabetes, and 2 (11.8%) reported diagnoses of prediabetes. In total, 16 of 17 (94.1%) participants reported currently taking one or more medication to manage their BP, with 12 (70.6%) participants reporting having experienced at least 1 adverse reaction to antihypertensive medication.

When asked about making lifestyle changes for HBP management, all 17 participants indicated that making changes required significant patient effort, with 16 (94.1%) having indicated that making these changes was important for managing BP. In total, 14 of 17 (82.4%) participants indicated that patient input was important when lifestyle change counseling is provided for BP control. Furthermore, 10 of 17 (58.8%) participants indicated that they often do not implement lifestyle changes for BP control. The majority of interviewees (88.3%) would be “extremely” or “somewhat” comfortable using a smartphone app, patient portal, or computer program that could make recommendations for treatment. The vast majority (88.9%) also felt it “extremely” or “somewhat likely” that the tool would improve patient outcomes.

**Table 1:** General characteristics of surveyed populations.

Demographic characteristics			Survey-guided interview participants (n=17)		Survey participants (N=519)
Female sex, n (%)			9 (52.9)		260 (50.1)
Age (years), mean (SD)			69.2 (9.1)		41.2 (14.7)
**Age groups (years), n (%)**					
	18-39	0 (0)		272 (52.4)	
	40-59	4 (23.5)		172 (33.1)	
	60-69	3 (17.7)		46 (8.9)	
	70-79	8 (47.1)		26 (5.0)	
	80+	2 (11.8)		3 (0.6)	
**Race and ethnicity, n (%)**					
	White	17 (100)		343 (65.0)	
	African American	0 (0)		93 (17.6)	
	Asian	0 (0)		23 (4.4)	
	Latino or Hispanic	0 (0)		50 (9.5)	
**Related conditions, n (%)**					
	Diabetes	3 (17.7)		153 (26.2)	
	Prediabetes	2 (11.8)		90 (15.4)	
	Heart failure	1 (5.9)		47 (8.1)	
	Chronic kidney disease	0 (0)		33 (5.7)	
	Past myocardial infarction	0 (0)		79 (15.2)	
	Past stroke	0 (0)		58 (11.2)	
	Have ASCVD^a^	6 (35.3)		119 (22.9)	
Age with ASCVD (years), mean (SD)			75.5 (4.8)		41.1 (14.8)
Age without ASCVD (years), mean (SD)			64.8 (8.7)		41.2 (14.7)
**Antihypertensive medications, n (%)**					
	None	1 (5.9)		101 (19.5)	
	1	3 (17.6)		190 (36.6)	
	2	5 (29.5)		128 (24.7)	
	3	6 (35.3)		61 (11.8)	
	4	2 (11.8)		31 (6.0)	
**Blood pressure goals and control, n (%)**					
	Participants with a blood pressure goal	17 (100)		427 (82.3)	
	Participants who chose a goal in consultation with a doctor	7 (41.2)		276 (64.6)	
	Participants who have monitored blood pressure at home	17 (100)		426 (82.1)	
	Participants who were satisfied with blood pressure control	13 (76.5)		383 (73.8)	
	Participants for whom blood pressure control was a high priority	16 (94.1)		429 (82.7)	
**Comfort with decision support systems, n (%)**					
	Extremely comfortable	8 (47.1)		195 (37.6)	
	Somewhat comfortable	7 (41.2)		211 (40.7)	
	Neither comfortable nor uncomfortable	0 (0)		90 (17.3)	
	Somewhat uncomfortable	2 (11.8)		17 (3.3)	
	Extremely uncomfortable	0 (0)		6 (1.2)	

^a^ASCVD: atherosclerotic cardiovascular disease.

### Patient Survey

In all, 541 participants completed the survey. We excluded 22 incomplete responses. The 519 remaining responses are summarized in Table 1. Demographically, 260 (50.1%) participants identified as female, with a much younger mean age of 41.2 years as compared to the interviewees (69.2 years). The majority of participants (n=272, 52.4%) were younger than 40 years, with only 75 (14.5%) participants aged 60 years or older. We compared the groups (Multimedia Appendix 3 and Table 1) and noted a higher burden of disease in the younger adults than is usually reported. A majority (n=343, 65.0%) of participants identified as White.

These participants reported generally good HBP control and 73.8% (383/519) of them were satisfied with the control. Most, 82.3% (427/519) of participants, reported having a specific BP goal, with 64.6% (276/519) of those having selected that goal in consultation with a doctor. Overall, 82.1% (426/519) of participants reported having monitored their BP at home either currently or previously. A large majority, 82.7% (n=429) of participants, reported that controlling their BP was a high or very high priority.

Significant comorbidities were reported by these participants, with 26.2% (n=153) of them having reported a diabetes diagnosis, 15.4% (n=90) of them having reported a prediabetes diagnosis, 8.1% (n=47) of them having reported a heart failure diagnosis, and 5.7% (n=33) of them having reported a chronic kidney disease diagnosis. Major adverse cardiovascular events were also reported, with 15.2% (n=79) of them having reported a past heart attack and 11.2% (n=58) of them having reported a past stroke. Based on self-report of one of heart failure diagnosis, past myocardial infarction, or past stroke, we determined that 22.9% (119) of survey participants have ASCVD. A minority of patients, 19.5% (n=101) of them, reported taking no medication to manage their BP, while 36.6% (190) of them took one medication and 24.7% (n=128) of them took 2 medications.

Participants were asked to arrange lifestyle change recommendations for HBP management into their personal order of priority. Participants could only include quitting smoking or moderating alcohol intake in their priority list if they indicated that they currently smoked tobacco or drank alcohol, respectively.

**Figure 4:**
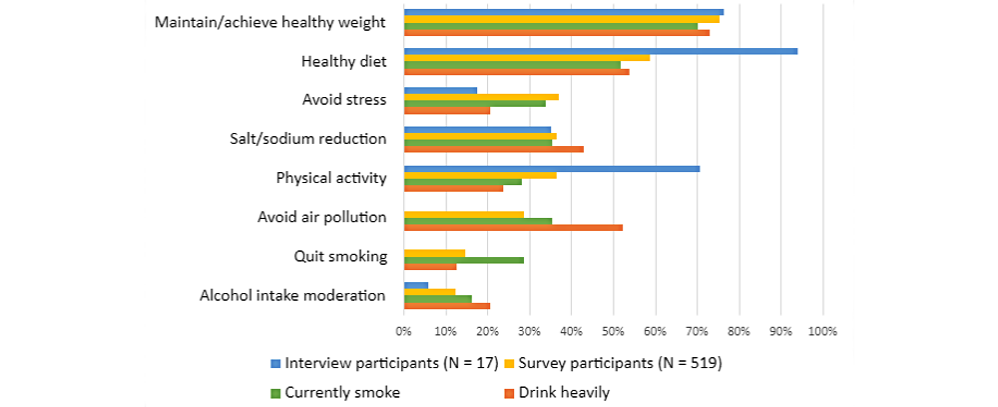
Percentage of participants' top 3 selections for lifestyle changes prioritized. Patients were considered to be drinking heavily if they consume >8 drinks per week if female and >15 drinks per week if male. The currently smoke category includes all current smokers, including those trying to quit.

As shown in Figure 4, we examined the top 3 selected priorities for each response. Maintaining or achieving a healthy weight was the most common priority among all participants (391/519, 75.3%) among the top 3 priorities, followed by eating a healthy diet (305/519, 58.7%). Among participants who currently smoke, only 28.7% (76/265) of them identified smoking cessation as one of their top 3 priorities. Among those who can be classified as drinking heavily, alcohol intake moderation was one of the top 3 priorities for 20.6% (13/63) of participants.

When asked about their comfort with using systems, such as smartphone apps, patient portals, and computer programs that provide recommendations based on a person’s BP history, 37.6% (195/519) of participants reported being “extremely comfortable,” with 40.7% (211/519) of them having reported that they were “somewhat comfortable.”

The first comparison (Figure 1) asked participants to consider whether a BP average display or a BP history chart was more trustworthy. The BP history chart was considered more trustworthy than the thermometer style display by 55.7% (289/519; 26.6%, sign test *P*=.002), though participants reported a similar likelihood to take action based on both charts: 80.9% (420/519) for average display versus 78.2% (406/519) for BP history chart (chi-square *P*=.28).

The second comparison (Figure 2) asked patients whether a display with lifestyle change goals prioritized by clinician priorities for hypertension treatment was more trustworthy than a display presenting lifestyle change goals without additional advice. Most participants, 60.9% (316/519), considered the display that included clinician advice more trustworthy, while 16.4% (85/519) of them considered the display without clinician prioritization more trustworthy (sign test, *P*<.001). Again, patients reported similar likelihood to take action based on both displays (*P*=.56).

The third comparison (Figure 3) asked participants to consider whether a display providing examples of specific potential antihypertensive medications that a doctor may prescribe and the guidelines supporting the use of those medications was more trustworthy than a display that advised patients to discuss medication options and potential side effects with their doctor. A majority of participants, 59.2% (307/519) considered the display that provided specific examples of medications and the supporting guidelines more trustworthy, while 18.5% (96/519) of them considered the latter display more trustworthy (*P*<.001). As with the other comparisons, participants reported similar likelihood to take action based on both displays (*P*=.77).

## Discussion

### Principal Findings

In both the interviews and the survey, we found favorable attitudes toward controlling BP through CDS applications. Most participants had monitored their BP at home and considered BP control a personal health priority. This experienced and motivated patient population spanned multiple demographics and indicates that there is a high perceived need for tools that better engage patients in hypertension care.

Survey participants had reported a high level of knowledge about BP goal setting. They also indicated a preference for more complete information presentation, including information about BP history, clinician-endorsed goals, and potential pharmacologic treatments for hypertension. Social or relational information, such as what clinicians would recommend or what other patients would do, was deemed particularly trustworthy.

Participants reported weight management and healthy dieting as their top priorities for lifestyle change-related goal setting. Among smokers and those who drink heavily, smoking cessation and alcohol intake moderation, 2 interventions that are known to help reduce both BP and ASCVD risk, were not highly prioritized. This is an opportunity for CDS tools to encourage patient goal setting by presenting these options as suggested priorities, as patients indicated receptiveness to suggested prioritization of lifestyle changes in A/B testing.

### Limitations

This research has several limitations. A major concern is that the population was not representative of those with HBP in the United States. The population was skewed toward younger, more technologically literate, and was less representative of underserved communities. This is an issue to be addressed in future work: to understand how better to engage these communities. Self-reported comorbidities require good health literacy to be accurate; our prior studies have shown reasonable accuracy in this group [31]. The rate of heart attack and stroke among adults younger than 40 years was much higher than expected; however, this group is growing rapidly [3]. Future surveys may address these concerns through health literacy screening and by stratifying survey participant subpopulations to achieve overall distributions closer to the population. Future versions of the tool could be created by engaging users historically marginalized by health care in a human-centered design process.

### Comparison With Prior Work and Future Research Needs

CDS interventions require engagement by different stakeholders. CDS can remind patients of their goals and promote adherence to those goals. Our survey results suggest that patients perceive they would act on information and recommendations displayed by the tool; however, significant previous work has shown that people overestimate their own actions [32]. By using displays that provide patients with more complete information about their BP history and options for goal setting and treatment, patients may better trust the recommendations provided by the tool. Providers have high fatigue with alerts, but may be able to transfer trust in CDS to patients, as we found in a previous survey [33]. Given the high priority that patients in the survey assigned to HBP management, CDS tools may be used to better engage patients in shared decision-making with their care team.

Engaging patients with these tools continues to be a challenge, however. Substantial work to understand how to engage patients, especially those who are historically underserved, has been undertaken, but disparities remain [34-36]. Additions of coaching and other supports may help key populations engage in CDS [15]. Similarly, improving the visualizations of the data and the manner in which recommendations are delivered was identified by patients as being important to engage in the CDS. Previous work has highlighted the importance of simple, clear, consistent design in CDS tools; the apparent contradiction of wanting more information and having limited attention make acting on these suggestions more difficult. Rapid cycle testing may help resolve these contradictions.

### Conclusions

Overall, this digitally literate group of patients was ready to engage with CDS tools and provided substantial guidance on the optimization of these tools through meaningful visualizations with context provided through evidence and from trusted groups. Next steps include expanding the population to those with lower digital literacy and testing the visualizations, reminders, and tailored messages in the real world through a pragmatic trial.

## Appendix

Multimedia Appendix 1 Hypertension patient interview questionnaire.

Multimedia Appendix 2 Hypertension patient survey (revised).

Multimedia Appendix 3 These are the data for the survey, de-identified, and with specific worksheets for the breakdown of key populations as requested.

## Abbreviations

ASCVD: atherosclerotic cardiovascular disease
BP: blood pressure
CDS: clinical decision support
HBP: high blood pressure
IRB: institutional review board
