# Supplementary material for: Patient-Facing Clinical Decision Support for High Blood Pressure Control: Patient Survey
Source: JMIR Cardio. 2023 Jan 23;7:e39490. doi: 10.2196/39490 (PMC9903181; doi:10.2196/39490)
Supplement: Multimedia Appendix 1 [file cardio_v7i1e39490_app1.pdf]

Hypertension Patient Interview Guide

Start of Block: General Questions

Hypertension Interview

This interview has been developed by the Care Management Plus Team at Oregon Health & Science University to obtain patient input on treatment strategies for high blood pressure (hypertension). Based on your feedback, we hope to better understand how to provide information to future patients and care team personnel, as well as to obtain a better understanding of patient preferences for high blood pressure management and treatment. Our goal is to develop a clinical decision support tool to aid patients and care team members in making informed care decisions regarding high blood pressure.

In this interview, you will be asked questions related to your history of high blood pressure, how it has been treated, how you would like to treat it, and what you would recommend for fictional patients with high blood pressure. Please answer the questions according to your own experience and preferences.

Page Break

How do you identify?

- ○ Female (1)
- ○ Male (2)
- ○ Other (6) ____
- ○ Prefer not to answer (5)

What is your age?

____

Please specify your race and ethnicity.
Check all that apply

- ☐ White / Caucasian (1)
- ☐ Black / African-American (2)
- ☐ Latino / Hispanic (8)
- ☐ Asian (3)
- ☐ Native American (4)
- ☐ Native Hawaiian / Pacific Islander (5)
- ☐ Two or More (9)
- ☐ Other / Unknown (6)
- ☐ I prefer not to say (7)

How high a priority is controlling your blood pressure?

- ○ Very high (1)
- ○ High (2)
- ○ Moderate (3)
- ○ Low (4)
- ○ Not important (5)

How satisfied have you been with your ability to control your blood pressure?

- ○ Very satisfied (1)
- ○ Somewhat satisfied (2)
- ○ Neither satisfied nor dissatisfied (3)
- ○ Somewhat dissatisfied (4)
- ○ Very dissatisfied (5)

Thank you. Now we would like to know about your experience with health technology.

How comfortable are you using smartphone apps for health purposes?

- ◯ Extremely comfortable (18)
- ◯ Somewhat comfortable (19)
- ◯ Neither comfortable nor uncomfortable (20)
- ◯ Somewhat uncomfortable (21)
- ◯ Extremely uncomfortable (22)
- ◯ Don't know (23)

How comfortable are you using patient portals, such as MyChart?

- ◯ Extremely comfortable (18)
- ◯ Somewhat comfortable (19)
- ◯ Neither comfortable nor uncomfortable (20)
- ◯ Somewhat uncomfortable (21)
- ◯ Extremely uncomfortable (22)
- ◯ Don't know (24)

End of Block: General Questions

Start of Block: Health/Graph Literacy

Please answer the following questions to the best of your ability, using the provided graphics.

Medication label 1: If you take your first tablet at 7:00 am, when should you take the next one?

____

Medication label 1: And the next one after that?

____

Medication label 2: If you have a lunch at noon and plan to take the medication **BEFORE a meal**, what time do you have to take the medication?

____

Medication label 2: If you have a lunch at noon and plan to take the medication **AFTER a meal**, what time do you have to take the medication?

____

Your blood pressure today is 140/100. Is this normal?

- ○ Yes (1)
- ○ No (2)
- ○ Unsure (3)

Appointment slip: When is the appointment date?

____

Appointment slip: Where will the appointment take place?

____

If you eat one serving of ramen, how many calories will you eat?

____

If you are allowed to eat 2,400 milligrams of sodium per day, how many servings of ramen could you have?

____

Your doctor advises you to reduce the amount of saturated fat in your diet. If you decide not to eat a serving of ramen today, how many grams of saturated fat would you be reducing?

____

If you usually eat 2,900 calories in a day, what percentage of your daily value of calories will you be eating if you eat one serving of this ramen?

____

Pretend that you are allergic to the following substances: MSG, wheat, shrimp, and honey: Is it safe for you to eat this ramen?

○ Yes (1)

○ No (2)

End of Block: Health/Graph Literacy

Start of Block: Blood Pressure Monitoring

What is your own blood pressure goal?

○ ≤ 150/90 mmHg (6)

○ ≤ 140/90 mmHg (5)

○ ≤ 135/85 mmHg (3)

○ ≤ 130/80 mmHg (4)

○ < 120/80 mmHg (1)

○ I do not have a specific goal (2)

○ I do not know my goal (7)

Display This Question:

If What is your own blood pressure goal? != I do not have a specific goal

And What is your own blood pressure goal? != I do not know my goal

How did you select this goal?

- My doctor chose it for me (1)
- I chose it during or after meeting with my doctor at an appointment (5)
- I chose it based on what I have read online (2)
- I chose it after consulting another source (3) ____
- Other (4) ____

Page Break

Have you monitored your blood pressure outside of your doctor's office?

- [ ] Yes, I have monitored my own blood pressure (1)
- [x] No, I have not monitored my own blood pressure (3)
- [ ] Unsure (2)

Display This Question:

If Have you monitored your blood pressure outside of your doctor's office? = Yes, I have monitored my own blood pressure

While monitoring your own blood pressure, how frequently are you supposed to take readings?

- ○ Four or more times per day (1)
- ○ Three times per day (2)
- ○ Twice per day (3)
- ○ Once per day (4)
- ○ Less than once per day (5)
- ○ I don't know (6)

Display This Question:

If Have you monitored your blood pressure outside of your doctor's office? = Yes, I have monitored my own blood pressure

How frequently do you check your blood pressure?

- ◯ Four or more times per day (1)
- ◯ Three times per day (2)
- ◯ Twice per day (3)
- ◯ Once per day (4)
- ◯ Less than once per day (5)

Display This Question:

If Have you monitored your blood pressure outside of your doctor's office? = Yes, I have monitored my own blood pressure

Have you seen any changes in your blood pressure since you began using home blood pressure monitoring?

- ◯ Yes - It has increased (1)
- ◯ Yes - It has decreased (2)
- ◯ No - It has remained steady (3)
- ◯ Unsure (4)

Display This Question:

If Have you monitored your blood pressure outside of your doctor's office? = Yes, I have monitored my own blood pressure

Please walk us through your process to monitor your own blood pressure, starting when you get ready to when you are done.

____

____

____

____

____

Display This Question:

If Have you monitored your blood pressure outside of your doctor's office? = Yes, I have monitored my own blood pressure

How do you communicate your home blood pressure readings to your doctor?

- ○ My monitoring device automatically sends the information to my doctor (1)
- ○ I enter measurements into an online form or smartphone app and send that information to my doctor (2)
- ○ I keep a written list of blood pressure readings and mail / bring it to my doctor (3)
- ○ Other (4) ____

Display This Question:

If Have you monitored your blood pressure outside of your doctor's office? = No, I have not monitored my own blood pressure

If your doctor were to recommend that you monitor your blood pressure outside of office visits, how comfortable would you be doing so?

- ○ Extremely comfortable (18)
- ○ Somewhat comfortable (19)
- ○ Neither comfortable nor uncomfortable (20)
- ○ Somewhat uncomfortable (21)
- ○ Extremely uncomfortable (22)

How do you rate your level of comfort with using a smartphone app to share blood pressure results with your doctor?

- ○ Extremely comfortable (19)
- ○ Somewhat comfortable (20)
- ○ Neither comfortable nor uncomfortable (21)
- ○ Somewhat uncomfortable (22)
- ○ Extremely uncomfortable (23)

How confident are you that using a smartphone app to share your blood pressure results with your care team will help you manage your blood pressure?

- ○ Very confident (32)
- ○ Somewhat confident (33)
- ○ Neither confident nor not confident (34)
- ○ Probably not confident (35)
- ○ Not confident at all (36)

End of Block: Blood Pressure Monitoring

Start of Block: Lifestyle Questions

Since being diagnosed with Hypertension, has your doctor provided guidance regarding lifestyle changes?
Check all that apply

- ▢ Yes, my doctor has counseled me about my diet (1)
- ▢ Yes, my doctor has counseled me about lowering my salt and/or sodium intake (2)
- ▢ Yes, my doctor has counseled me about increased physical activity (3)
- ▢ Yes, my doctor has counseled me about weight loss (4)
- ▢ Yes, my doctor has counseled me about stress reduction (6)
- ▢ Yes, my doctor has counseled me about avoiding exposure to air pollution (7)
- ▢ ⊗No, I have not been counseled about any of these changes (5)

Display This Question:

If Since being diagnosed with Hypertension, has your doctor provided guidance regarding lifestyle ch... = Yes, my doctor has counseled me about my diet

Have you made any changes to your diet since your doctor gave you counseling?

- ○ I have made major dietary changes (1)
- ○ I have made moderate dietary changes (2)
- ○ I have made minor dietary changes (3)
- ○ I have not made dietary changes (4)
- ○ I initially made dietary changes but have not maintained those changes (5)

Display This Question:

If Since being diagnosed with Hypertension, has your doctor provided guidance regarding lifestyle ch... = Yes, my doctor has counseled me about lowering my salt and/or sodium intake

Have you lowered your salt and/or sodium intake?

◯ Yes (1)

◯ No (2)

Display This Question:

If Since being diagnosed with Hypertension, has your doctor provided guidance regarding lifestyle ch... = Yes, my doctor has counseled me about increased physical activity

Have you made any changes to your physical activity since your doctor gave you counseling?

◯ I have become much more physically active (1)

◯ I have become somewhat more physically active (2)

◯ I have become slightly more physically active (3)

◯ I have not become more physically active (4)

◯ I initially became more physically active but have not maintained that change (5)

Display This Question:

If Since being diagnosed with Hypertension, has your doctor provided guidance regarding lifestyle ch... = Yes, my doctor has counseled me about weight loss

Have you made any progress towards reaching your target weight?

◯ Yes (1)

◯ No (2)

◯ Unsure (3)

Display This Question:

If Since being diagnosed with Hypertension, has your doctor provided guidance regarding lifestyle ch... = Yes, my doctor has counseled me about stress reduction

Have you taken any steps to reduce stress?

- ◯ Yes (1) ____
- ◯ No (2)
- ◯ Unsure (3)

Display This Question:

If Since being diagnosed with Hypertension, has your doctor provided guidance regarding lifestyle ch... = Yes, my doctor has counseled me about avoiding exposure to air pollution

Have you taken any steps to reduce your exposure to air pollution?

- ◯ Yes (1) ____
- ◯ No (2)
- ◯ Unsure (3)

Do you smoke?

- ◯ I currently smoke tobacco such as cigarettes or cigars (1)
- ◯ I currently smoke with a vaping device (5)
- ◯ I currently smoke tobacco or vape and am trying to quit (2)
- ◯ I used to smoke tobacco or vape, but have quit (3)
- ◯ I have never smoked tobacco or vaped (4)

Display This Question:

If Do you smoke? = I currently smoke tobacco such as cigarettes or cigars

Or Do you smoke? = I currently smoke with a vaping device

Or Do you smoke? = I currently smoke tobacco or vape and am trying to quit

Have you been counseled by your doctor about quitting smoking?

- Yes (1)
- No (2)

How much alcohol do you normally drink?

- Fifteen or more drinks per week (1)
- Eight to fourteen drinks per week (2)
- Five to seven drinks per week (3)
- One to four drinks per week (4)
- Less than one drink per week (6)
- I don't drink (5)

Display This Question:

If How much alcohol do you normally drink? != I don't drink

Have you been counseled by your doctor about lowering your alcohol intake?

- Yes (1)
- No (2)

Please arrange your personal health priorities from top to bottom:
_______ Reaching / maintaining a healthy body weight (1)
_______ Avoiding exposure to air pollution (8)
_______ Maintaining a healthy diet (2)
_______ Reducing sodium intake / maintaining a low sodium intake (3)
_______ Getting enough exercise (4)

How much alcohol do you normally drink? != I don't drink

_______ Lowering alcohol intake (5)

Do you smoke? != I used to smoke tobacco or vape, but have quit

And Do you smoke? != I have never smoked tobacco or vaped

_______ Quitting smoking (6)
_______ Reducing/avoiding stress (7)

Has your doctor recommended you talk with a dietitian to discuss healthy eating habits for managing high blood pressure?

- ◯ My doctor has recommended I see a dietitian (4)
- ◯ My doctor has not recommended I see a dietitian (5)
- ◯ I do not remember (3)

Display This Question:

If Has your doctor recommended you talk with a dietitian to discuss healthy eating habits for managi... = My doctor has recommended I see a dietitian

Have you seen a dietitian to discuss healthy eating habits to help manage high blood pressure?

- ◯ I have talked to a dietitian (1)
- ◯ I intend to talk to a dietitian (2)
- ◯ I have not talked to a dietitian (3)
- ◯ I do not remember (4)

In general, how much effort are you willing to give for making lifestyle changes?

- ◯ Significant, daily effort (1)
- ◯ A moderate effort (2)
- ◯ A little effort (3)
- ◯ Nothing that changes my daily routine (4)
- ◯ None (5)
- ◯ Unsure (6)

How do you rate your level of comfort with using a smartphone app to help you make lifestyle changes?

- ◯ Extremely comfortable (24)
- ◯ Somewhat comfortable (25)
- ◯ Neither comfortable nor uncomfortable (26)
- ◯ Somewhat uncomfortable (27)
- ◯ Extremely uncomfortable (28)

Would using a smartphone app that tracks your lifestyle goals help you make those changes?

- ◯ Definitely yes (27)
- ◯ Probably yes (28)
- ◯ Might or might not (29)
- ◯ Probably not (30)
- ◯ Definitely not (31)

If an app or tool suggested that you make some or all of these lifestyle changes in your day to day life, would you be comfortable acting on its recommendations?

	Yes (1)	No (2)	Unsure (4)
Increasing physical activity (1)	◯	◯	◯
Making dietary changes (2)	◯	◯	◯
Reducing salt/sodium intake (3)	◯	◯	◯
Losing weight (4)	◯	◯	◯
Do you smoke? = I currently smoke tobacco such as cigarettes or cigars Or Do you smoke? = I currently smoke with a vaping device Stopping smoking (5)	◯	◯	◯
How much alcohol do you normally drink? != I don't drink Reducing alcohol intake (6)	◯	◯	◯
Reducing stress (7)	◯	◯	◯
Avoiding exposure to air pollution (8)	◯	◯	◯

Can you tell us why you would or would not be comfortable acting on the app or tool's recommendations?

____

____

End of Block: Lifestyle Questions

Start of Block: Comorbidities

Have you been diagnosed with any of the following medical conditions?
Choose all that apply. If none apply, leave this question blank.

- ☐ Diabetes (1)
- ☐ Prediabetes (6)
- ☐ Chronic kidney disease (CKD) (2)
- ☐ Heart failure (3)
- ☐ Unsure (7)

Have you previously had a heart attack?

- ○ Yes (1)
- ○ No (2)
- ○ Unsure (3)

Have you previously had a stroke?

- ○ Yes (1)
- ○ No (2)
- ○ Unsure (3)

End of Block: Comorbidities

Start of Block: Adverse Event History

Can you tell us what types of medication you take to manage your blood pressure?
Check all that apply

▢ **Angiotensin-converting enzyme (ACE) - Inhibitor.** ACE-Inhibitors include Capoten (captopril), Vasotec (enalapril), Prinivil, Zestril (lisinopril), Lotensin (benazepril), Monopril (fosinopril), Altace (ramipril), Accupril (quinapril), Aceon (perindopril), Mavik (trandolapril), and Univasc (moexipril). (1) ____

▢ **Angiotensin II Receptor Blocker (ARB).** ARBs include Atacand (candesartan), Avapro (irbesartan), Benicar (olmesartan), Cozaar (losartan), Diovan (valsartan), Micardis (telmisartan), and Teveten (eprosartan). (2) ____

▢ **Thiazide-type diuretic.** Thiazide-type diuretics include Chlorothiazide (Diuril), Chlorthalidone, Hydrochlorothiazide (Microzide), Indapamide, and Metolazone. (3) ____

▢ **Calcium Channel Blocker.** CCBs include Norvasc (amlodipine), Plendil (felodipine), DynaCirc (isradipine), Cardene (nicardipine), Procardia XL, Adalat (nifedipine), Cardizem, Dilacor, Tiazac, Diltia XL (diltiazem), Sular (Nisoldipine), Isoptin, Calan, Verelan, and Covera-HS (verapamil). (4) ____

▢ **β-Blocker.** Beta-blockers include Acebutolol (Sectral), Atenolol (Tenormin), Betaxolol (Kerlone), Bisoprolol (Zebeta, Ziac), Carteolol (Cartrol), Carvedilol (Coreg), Labetalol (Normodyne, Trandate), Metoprolol (Lopressor, Toprol-XL), Nadolol (Corgard), Nebivolol (Bystolic), Penbutolol (Levatol), Pindolol (Visken), Propanolol (Inderal), Sotalol (Betapace), and Timolol (Blocadren). (5) ____

▢ Another type of antihypertensive (6) ____

▢ Unsure (8) ____

☒ None (7)

Have you experienced any adverse drug events from high blood pressure medication? Check all that apply

- ☐ Low blood pressure (1)
- ☐ Dizziness (2)
- ☐ Falls (3)
- ☐ Constipation (4)
- ☐ Nausea (5)
- ☐ Cough (14)
- ☐ Fatigue (6)
- ☐ Hair loss (7)
- ☐ Drowsiness (8)
- ☐ Low red/white/platelet blood counts (9)
- ☐ Skin reactions (10)
- ☐ Loss of appetite (11)
- ☐ Diarrhea (12)
- ☐ Edema (16)
- ☐ Low potassium levels (17)
- ☐ Low sodium levels (18)
- ☐ Other (13) ____
- ☒ No (15)

How did you respond to any of the event(s) in the previous question?
Check all that apply

- ☐ I called my doctor (1)
- ☐ I went to see my doctor (2)
- ☐ I went to the emergency room (3)
- ☐ I recorded the event in MyChart or my patient portal (4)
- ☐ I waited for the event to pass (5)
- ☐ I called my family and/or friends (7)
- ☐ I searched for answers online (8)
- ☐ Other (6) ____

How difficult is it to follow your schedule of medications while maintaining your daily routine?

- ○ Very difficult (1)
- ○ Somewhat difficult (2)
- ○ Neither easy nor difficult (3)
- ○ Somewhat easy (4)
- ○ Very easy (5)

How comfortable would you be with using a smartphone app that tracks your schedule of medications and reminds you when to take them?

- ○ Extremely comfortable (36)
- ○ Somewhat comfortable (37)
- ○ Neither comfortable nor uncomfortable (38)
- ○ Somewhat uncomfortable (39)
- ○ Extremely uncomfortable (40)

How comfortable would you be with using this smartphone app if it also informed your doctor when you take your medications?

- ○ Extremely comfortable (35)
- ○ Somewhat comfortable (36)
- ○ Neither comfortable nor uncomfortable (37)
- ○ Somewhat uncomfortable (38)
- ○ Extremely uncomfortable (39)

How comfortable would you be with using a smartphone app to report adverse events?

- ○ Extremely comfortable (25)
- ○ Somewhat comfortable (26)
- ○ Neither comfortable nor uncomfortable (27)
- ○ Somewhat uncomfortable (28)
- ○ Extremely uncomfortable (29)

Would an app that allows you to track medications and report reactions to the medication be helpful to you?

- Definitely yes (11)
- Probably yes (12)
- Might or might not (13)
- Probably not (14)
- Definitely not (15)

End of Block: Adverse Event History

Start of Block: Case Study

For this section, imagine you are a doctor seeing patients in a clinic.

A 40-year-old patient with high blood pressure arrives at your clinic. Their recent blood pressure readings are as follows:

Do you want to see a graph of these readings? The average values?

- Display the graph (1)
- Display the average values (2)

Display This Question:

If For this section, imagine you are a doctor seeing patients in a clinic. A 40-year-old patient w... = Display the graph

---

Display This Question:

If For this section, imagine you are a doctor seeing patients in a clinic.   A 40-year-old patient w... = Display the average values

Average SBP: 144.5 mmHg

Average DBP: 86.93 mmHg

This patient is not currently taking antihypertensive medication. The patient is overweight, has a high pulse rate, drinks heavily, and smokes a pack of cigarettes each day.

Please drag and drop the following interventions into your order of priority for the purpose of lowering the patient's blood pressure.
_______ Recommend weight loss (1)
_______ Recommend salt restriction and sodium intake reduction (3)
_______ Recommend moderating alcohol consumption (4)
_______ Recommend diet change, focused on fruit and vegetable intake and fat restriction (2)
_______ Recommend increased physical activity (5)
_______ Recommend smoking cessation (6)
_______ Recommend reducing stress (8)
_______ Recommend limiting exposure to air pollution (9)

Please rate the above lifestyle changes according to the following categories:

	1	2	3	4	5	6	7

How important is it to make these changes for the purpose of lowering blood pressure? 1 = Not important 7 = Extremely important ()	
How frequently do care teams recommend these changes? 1 = Never 7 = All the time ()	
How frequently do other people successfully make these lifestyle changes? 1 = Never 7 = All the time ()	
How much patient input is required to recommend these lifestyle changes? 1 = None 7 = Patient-driven ()	
How much patient effort is required to make these lifestyle changes? 1 = None 7 = Extensive, daily work ()	

If this patient were instructed to monitor their blood pressure at home twice per day, how likely do you think they would keep to that schedule?

- ◯ Extremely likely (18)
- ◯ Somewhat likely (19)
- ◯ Neither likely nor unlikely (20)
- ◯ Somewhat unlikely (21)
- ◯ Extremely unlikely (22)

Display This Question:

If If For this section, imagine you are a doctor seeing patients in a clinic.   A 40-year-old patient with high blood pressure arrives at your clinic. Their recent blood pressure readings are as f... q://QID65/SelectedChoicesCount Is Greater Than or Equal to 1

Which information display was most useful to you in making decisions about this patient?

- ○ The blood pressure history table (1)
- ○ The blood pressure history graph (2)
- ○ The blood pressure history average values (3)

End of Block: Case Study

Start of Block: Adverse Event Reporting

A 65-year-old Latino man has high blood pressure and stage 3 chronic kidney disease. He is on a diet for hypertension and takes lisinopril and hydrochlorothiazide to manage his blood pressure.

If this patient experienced occasional dizziness but did not fall, what should he do?
Check all that apply

- ▢ Stop taking his medication (1)
- ▢ Call his doctor (2)
- ▢ Go see his doctor (3)
- ▢ Record the event in MyChart or his patient portal (5)
- ▢ Sit down for a few minutes until the dizziness passes (6)
- ▢ Be careful going about his daily routine (7)
- ▢ Go to the emergency room (8)
- ▢ Other (9) ____

If this patient had diarrhea, what should he do?
Check all that apply

- ▢ Stop taking his medication (1)
- ▢ Call his doctor (2)
- ▢ Go see his doctor (3)
- ▢ Record the event in MyChart or patient portal (4)
- ▢ Rest and let it pass (5)
- ▢ Go to the emergency room (6)
- ▢ Other (8) ____

What should this patient do if his dizziness worsened and he fell twice?
Check all that apply

- ▢ Stop taking his medication (1)
- ▢ Call his doctor (2)
- ▢ Go see his doctor (3)
- ▢ Record the event in MyChart or patient portal (4)
- ▢ Go to the emergency room (5)
- ▢ Be careful going about his daily routine (7)
- ▢ Other (8) ____

End of Block: Adverse Event Reporting

Start of Block: Final Questions

Thank you for imagining that you are a doctor. In our last few questions, we would like to know about you

How high a priority is controlling your blood pressure?

- ○ It is a very high priority (1)
- ○ It is a high priority (2)
- ○ It is a moderate priority (3)
- ○ It is a low priority (4)
- ○ It is not a priority (5)

How satisfied are you with your progress in managing your blood pressure?

- ○ Very satisfied (1)
- ○ Somewhat satisfied (2)
- ○ Neither satisfied nor dissatisfied (3)
- ○ Somewhat dissatisfied (4)
- ○ Very dissatisfied (5)

If a system (such as a smartphone app, patient portal, or computer program) could make recommendations for treatment based on patterns in a person's blood pressure history, how comfortable would you be to use it?

- ◯ Extremely comfortable (35)
- ◯ Somewhat comfortable (36)
- ◯ Neither comfortable nor uncomfortable (37)
- ◯ Somewhat uncomfortable (38)
- ◯ Extremely uncomfortable (39)

How likely would it be for such a tool to improve patient outcomes?

- ◯ Extremely likely (18)
- ◯ Somewhat likely (19)
- ◯ Neither likely nor unlikely (20)
- ◯ Somewhat unlikely (21)
- ◯ Extremely unlikely (22)

End of Block: Final Questions
