# Supplementary material for: Patient-Facing Clinical Decision Support for High Blood Pressure Control: Patient Survey
Source: JMIR Cardio. 2023 Jan 23;7:e39490. doi: 10.2196/39490 (PMC9903181; doi:10.2196/39490)
Supplement: Multimedia Appendix 2 [file cardio_v7i1e39490_app2.docx]

OHSU High Blood Pressure Decision Support Patient Survey

Start of Block: General Questions

Q1.1
**CLINICAL RESEARCH CONSENT AND AUTHORIZATIONSUMMARY OF KEY INFORMATION ABOUT THIS STUDY**   **TITLE:** Translating Blood Pressure (BP) guidelines into patient-centered interoperable sharable Clinical Decision Support (CDS)
**IRB#:** 20522   **PRINCIPAL INVESTIGATOR:** David Dorr, MD, MS, FACMI       (503) 418-2387   You are being asked to join a research study. This consent form contains important information to help you decide if you want to join the study or not.   **PURPOSE:** The purpose of the study is to assess patient knowledge of hypertension and how to present information to patients with hypertension.   **DURATION:** Your participation in the study will consist of completing a single survey, which should take about 10 minutes.   **PROCEDURES:**  If you decide to participate, you will complete a single survey in Qualtrics which will ask you about your history of high blood pressure management and treatment, your preferences for care, your preferences for how information is presented to you, and what actions fictional patients should take to manage their blood pressure.   **RISKS:** This is a minimal-risk study. The only risk to you is that your high blood pressure status could be revealed, though we are not collecting any identifiable information from you.   **COMPENSATION:** You will be compensated the amount you agreed upon before you entered the survey.   **ALTERNATIVES:** This is a voluntary research study. You do not have to join the study. Even if you decide to join now, you can change your mind later.   **END OF CONSENT SUMMARY**

Do you consent to participate in this survey?

- Yes, I consent to participate in this survey (1)
- No, I do not consent to participate in this survey (2)

Skip To: End of Block If CLINICAL RESEARCH CONSENT AND AUTHORIZATION SUMMARY OF KEY INFORMATION ABOUT THIS STUDY   TITLE:... = No, I do not consent to participate in this survey

Q1.2 This survey was created by the Care Management Plus Team at Oregon Health & Science University. The goal of this survey is to help us better understand what patients prefer when making decisions about how to manage and treat their high blood pressure (hypertension). We will use your response to create a tool that helps patients make decisions to improve their health through better high blood pressure outcomes.

 
We will ask you some questions about your demographic information, your own history of managing your blood pressure, your preferences for treatment, and how you would respond to being presented with information about blood pressure in several different formats.

| Page Break |  |
| --- | --- |

Q1.3 Have you ever been diagnosed with High Blood Pressure (Hypertension)?

- Yes (1)
- No (2)

Skip To: End of Block If Have you ever been diagnosed with High Blood Pressure (Hypertension)? = No

| Page Break |  |
| --- | --- |

Q1.4 How do you identify?

- Female (1)
- Male (2)
- Other (6) __________________________________________________
- Prefer not to answer (5)

Q1.5 What is your age?

________________________________________________________________

Skip To: End of Block If Condition: What is your age? Is Less Than 18. Skip To: End of Block.

Skip To: End of Block If Condition: What is your age? Is Greater Than 85. Skip To: End of Block.

Q1.6 Please specify your race and ethnicity.
 Check all that apply

- ⊗White / Caucasian (1)
- Black / African-American (2)
- Latino / Hispanic (8)
- Asian (3)
- Native American (4)
- Native Hawaiian / Pacific Islander (5)
- Other / Unknown (6)
- ⊗I prefer not to say (7)

| Page Break |  |
| --- | --- |

Q1.7 Which of these categories best describes your total combined family income for the past 12 months?
 This should include income (before taxes) from all sources, wages, rent from properties, social security, disability and/or veteran's benefits, unemployment benefits, workman's compensation, help from relatives (including child payments and alimony), and so on.

- Less than $5,000 (1)
- $5,000 through $11,999 (2)
- $12,000 through $15,999 (3)
- $16,000 through $24,999 (4)
- $25,000 through $34,999 (5)
- $35,000 through $49,999 (6)
- $50,000 through $74,999 (7)
- $75,000 through $99,999 (8)
- $100,000 or greater (9)
- Don't know (10)
- Prefer not to answer (11)

| Page Break |  |
| --- | --- |

Q1.8 How satisfied are you with your ability to control your blood pressure?

- Very satisfied (1)
- Somewhat satisfied (2)
- Neither satisfied nor dissatisfied (3)
- Somewhat dissatisfied (4)
- Very dissatisfied (5)

End of Block: General Questions

Start of Block: Blood Pressure Monitoring

Q2.1 What is your personal blood pressure goal?

- ≤ 150/90 mmHg (6)
- ≤ 140/90 mmHg (5)
- ≤ 135/85 mmHg (3)
- ≤ 130/80 mmHg (4)
- < 120/80 mmHg (1)
- I do not have a specific goal (2)
- I do not know my goal (7)

| Page Break |  |
| --- | --- |

Display This Question:

If What is your personal blood pressure goal? = ≤ 150/90 mmHg

Or What is your personal blood pressure goal? = ≤ 140/90 mmHg

Or What is your personal blood pressure goal? = ≤ 135/85 mmHg

Or What is your personal blood pressure goal? = ≤ 130/80 mmHg

Or What is your personal blood pressure goal? = < 120/80 mmHg

Q2.2 How did you select this goal?

- In consultation with my doctor (1)
- Through internet research (2)
- Based on my own knowledge/experience (3)
- Other (4) __________________________________________________

Q2.3 Do you monitor your blood pressure at home?

- Yes, I monitor or have monitored my blood pressure at home (1)
- No, I have not monitored my blood pressure at home (3)

End of Block: Blood Pressure Monitoring

Start of Block: Lifestyle Questions

Q3.1 Do you smoke?

- I currently smoke tobacco such as cigarettes or cigars (1)
- I currently smoke with a vaping device (5)
- I currently smoke tobacco or vape and am trying to quit (2)
- I used to smoke tobacco or vape, but have quit (3)
- I have never smoked tobacco or vaped (4)

| Page Break |  |
| --- | --- |

Q3.2 How much alcohol do you normally drink in a week?

- Fifteen or more drinks per week (1)
- Eight to fourteen drinks per week (2)
- Five to seven drinks per week (3)
- One to four drinks per week (4)
- Less than one drink per week (6)
- I don't drink (5)

| Page Break |  |
| --- | --- |

Q3.3 Please arrange these health priorities from highest priority to lowest priority for you (1=highest priority; 8=lowest priority):

______ Reaching / maintaining a healthy body weight (1)

______ Avoiding exposure to air pollution (8)

______ Maintaining a healthy diet (2)

______ Reducing sodium intake / maintaining a low sodium intake (3)

______ Getting enough exercise (4)

Display This Choice:

If How much alcohol do you normally drink in a week? != I don't drink

______ Lowering alcohol intake (5)

Display This Choice:

If Do you smoke? != I used to smoke tobacco or vape, but have quit

And Do you smoke? != I have never smoked tobacco or vaped

______ Quitting smoking (6)

______ Reducing/avoiding stress (7)

End of Block: Lifestyle Questions

Start of Block: Comorbidities

Q4.1 Have you been diagnosed with any of the following medical conditions?
 Choose all that apply. If none apply, leave this question blank.

- Diabetes (1)
- Prediabetes (6)
- Chronic kidney disease (CKD) (2)
- Heart failure (3)
- Unsure (7)

| Page Break |  |
| --- | --- |

Q4.2 Have you ever had a heart attack?

- Yes (1)
- No (2)
- Unsure (3)

| Page Break |  |
| --- | --- |

Q4.3 Have you ever had a stroke?

- Yes (1)
- No (2)
- Unsure (3)

| Page Break |  |
| --- | --- |

Q4.4 How many medications do you take to manage your blood pressure?

- None (1)
- 1 (2)
- 2 (3)
- 3 (4)
- 4 or more (5)
- Unsure (6)

End of Block: Comorbidities

Start of Block: BP Visualizations

Q5.1 Please compare the following displays and consider how you would prefer the tool present information about your blood pressure.

| Page Break |  |
| --- | --- |

Q5.2 Display 1:

|  | 1 | 2 | 3 | 4 | 5 | 6 | 7 |
| --- | --- | --- | --- | --- | --- | --- | --- |

| Based on this information, how likely would you be to take action?(1 = very unlikely, 7 = very likely) () | 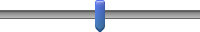 |
| --- | --- |

| Page Break |  |
| --- | --- |

Q5.3 Display 2:

|  | 1 | 2 | 3 | 4 | 5 | 6 | 7 |
| --- | --- | --- | --- | --- | --- | --- | --- |

| Based on this information, how likely would you be to take action?(1 = very unlikely, 7 = very likely) () | 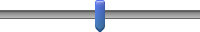 |
| --- | --- |

| Page Break |  |
| --- | --- |

Q5.4 What action, if any, would you take based on the information presented in these displays?
 Check all that apply

- Set a blood pressure goal in the tool (4)
- Contact your care team for more information (5)
- Get more information about blood pressure goals from the internet (6)
- Other (7) __________________________________________________
- ⊗None (8)

| Page Break |  |
| --- | --- |

Q5.5 Which display is more trustworthy?

|  | Display 1 | No Preference | Display 2 |
| --- | --- | --- | --- |

|  | 1 | 2 | 3 | 4 | 5 | 6 | 7 |
| --- | --- | --- | --- | --- | --- | --- | --- |

| Please tell us which display you trust more () | 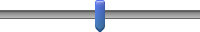 |
| --- | --- |

End of Block: BP Visualizations

Start of Block: Goal Visualizations

Q6.1 Please compare the following displays and consider how much guidance you would like the tool to provide when initially setting personal goals to help you manage your blood pressure.

| Page Break |  |
| --- | --- |

Q6.2 Display 1:

|  | 1 | 2 | 3 | 4 | 5 | 6 | 7 |
| --- | --- | --- | --- | --- | --- | --- | --- |

| Based on this information, how likely would you be to take action? (1 = very unlikely, 7 = very likely) () | 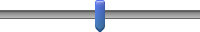 |
| --- | --- |

| Page Break |  |
| --- | --- |

Q6.3 Display 2:

|  | 1 | 2 | 3 | 4 | 5 | 6 | 7 |
| --- | --- | --- | --- | --- | --- | --- | --- |

| Based on this information, how likely would you be to take action? (1 = very unlikely, 7 = very likely) () | 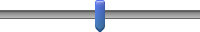 |
| --- | --- |

| Page Break |  |
| --- | --- |

Q6.4 What action, if any, would you take based on the information presented in these displays?
 Check all that apply

- Set a goal for smoking cessation, dietary change, or physical activity (4)
- Contact your care team for more information (5)
- Get more information about goals from the internet (6)
- Other (7) __________________________________________________
- ⊗None (8)

| Page Break |  |
| --- | --- |

Q6.5 Which display is more trustworthy?

|  | Display 1 | No Preference | Display 2 |
| --- | --- | --- | --- |

|  | 1 | 2 | 3 | 4 | 5 | 6 | 7 |
| --- | --- | --- | --- | --- | --- | --- | --- |

| Please tell us which display you trust more () | 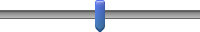 |
| --- | --- |

End of Block: Goal Visualizations

Start of Block: Pharmacology Information

Q7.1 Please compare the following displays for a patient diagnosed with hypertension who is not currently on hypertensive treatment. We would like you to consider how much information the tool should provide if you were that patient.

| Page Break |  |
| --- | --- |

Q7.2 Display 1:

|  | 1 | 2 | 3 | 4 | 5 | 6 | 7 |
| --- | --- | --- | --- | --- | --- | --- | --- |

| Based on this information, how likely would you be to take action? (1 = very unlikely, 7 = very likely) () | 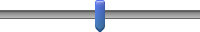 |
| --- | --- |

| Page Break |  |
| --- | --- |

Q7.3 Display 2:

|  | 1 | 2 | 3 | 4 | 5 | 6 | 7 |
| --- | --- | --- | --- | --- | --- | --- | --- |

| Based on this information, how likely would you be to take action? (1 = very unlikely, 7 = very likely) () | 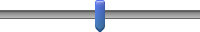 |
| --- | --- |

| Page Break |  |
| --- | --- |

Q7.4 What action, if any, would you take based on the information presented in these displays?
 Check all that apply

- Learn more about the guidelines supporting these recommendations (4)
- Contact your care team for more information (5)
- Get more information about antihypertensive medications from the internet (6)
- Other (7) __________________________________________________
- ⊗None (8)

| Page Break |  |
| --- | --- |

Q7.5 Which display is more trustworthy?

|  | Display 1 | No Preference | Display 2 |
| --- | --- | --- | --- |

|  | 1 | 2 | 3 | 4 | 5 | 6 | 7 |
| --- | --- | --- | --- | --- | --- | --- | --- |

| Please tell us which display you trust more () | 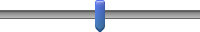 |
| --- | --- |

End of Block: Pharmacology Information

Start of Block: Case Study

Q8.1
For this section, imagine you are a doctor seeing patients in a clinic.
 
A 40-year-old patient with high blood pressure arrives at your clinic. Their recent blood pressure readings are as follows:
 

 Would you want to see a graph of these readings or see the average value of these readings?

- Display the graph (1)
- Display the average values (2)
- Keep it displayed as is (3)

| Page Break |  |
| --- | --- |

Display This Question:

If For this section, imagine you are a doctor seeing patients in a clinic.   A 40-year-old patient w... = Display the graph

Q8.2

| Page Break |  |
| --- | --- |

Display This Question:

If For this section, imagine you are a doctor seeing patients in a clinic.   A 40-year-old patient w... = Display the average values

Q8.3 Average SBP(systolic blood pressure): 144.5 mmHg

Average DBP (diastolic blood pressure): 86.93 mmHg

Q8.4 This patient is not currently taking any medication for their blood pressure. The patient is overweight, has a high pulse rate, drinks heavily, and smokes a pack of cigarettes each day.


Please drag and drop the following interventions into your order of priority for lowering this patient's blood pressure (1=highest priority; 8=lowest priority).

______ Recommend weight loss (1)

______ Recommend salt restriction and sodium intake reduction (3)

______ Recommend moderating alcohol consumption (4)

______ Recommend diet change, focused on fruit and vegetable intake and fat restriction (2)

______ Recommend increased physical activity (5)

______ Recommend smoking cessation (6)

______ Recommend reducing stress (8)

______ Recommend limiting exposure to air pollution (9)

| Page Break |  |
| --- | --- |

Q8.5 If this patient were instructed to monitor their blood pressure at home twice per day, is it likely that they would keep to that schedule?

- Extremely likely (18)
- Somewhat likely (19)
- Neither likely nor unlikely (20)
- Somewhat unlikely (21)
- Extremely unlikely (22)

| Page Break |  |
| --- | --- |

Display This Question:

If For this section, imagine you are a doctor seeing patients in a clinic.   A 40-year-old patient w... = Display the graph

Or For this section, imagine you are a doctor seeing patients in a clinic.   A 40-year-old patient w... = Display the average values

Q8.6 Which information display was most useful to you in making decisions about this patient?

- The blood pressure history table (1)
- The blood pressure history graph (2)
- The blood pressure history average values (3)

| Page Break |  |
| --- | --- |

Q8.7 A 65-year-old Latino man has high blood pressure and stage 3 chronic kidney disease. He is on a diet for hypertension and takes two medications to manage his blood pressure.

 If this patient experienced occasional dizziness but did not fall, what should he do?
Check all that apply

- Stop taking his medication (1)
- Call his doctor (2)
- Go see his doctor (3)
- Record the event in MyChart or his patient portal (5)
- Sit down for a few minutes until the dizziness passes (6)
- Be careful going about his daily routine (7)
- Go to the emergency room (8)
- Other (9) __________________________________________________

| Page Break |  |
| --- | --- |

Q8.8 What should this patient do if his dizziness worsened and he fell twice?
 Check all that apply

- Stop taking his medication (1)
- Call his doctor (2)
- Go see his doctor (3)
- Record the event in MyChart or patient portal (4)
- Go to the emergency room (5)
- Be careful going about his daily routine (7)
- Other (8) __________________________________________________

End of Block: Case Study

Start of Block: Final Questions

Q9.1
Thank you for imagining that you are a doctor. In our last few questions, we would like to know about you.

How high a priority is controlling your own blood pressure?

- It is a very high priority (1)
- It is a high priority (2)
- It is a moderate priority (3)
- It is a low priority (4)
- It is not a priority (5)

| Page Break |  |
| --- | --- |

Q9.2 If a system (such as a smartphone app, patient portal, or computer program) could make recommendations for treatment based on patterns in a person's blood pressure history, how comfortable would you be to use it?

- Extremely comfortable (35)
- Somewhat comfortable (36)
- Neither comfortable nor uncomfortable (37)
- Somewhat uncomfortable (38)
- Extremely uncomfortable (39)

| Page Break |  |
| --- | --- |

Q9.3 Is there anything else you would like us to know about your blood pressure?

________________________________________________________________

________________________________________________________________

________________________________________________________________

________________________________________________________________

________________________________________________________________

End of Block: Final Questions
